# Improved Dual Network Model for Aging of Rubber Composites under Set Strains

**DOI:** 10.1021/acs.macromol.3c01131

**Published:** 2023-08-22

**Authors:** Aaron M. Duncan, Keizo Akutagawa, Julien L. Ramier, James J. C. Busfield

**Affiliations:** † School of Engineering and Material Science, Queen Mary University of London, Mile End Road, London E1 4NS, U.K.; ‡ SLB Cambridge Research, Cambridge CB3 0EL, U.K.

## Abstract

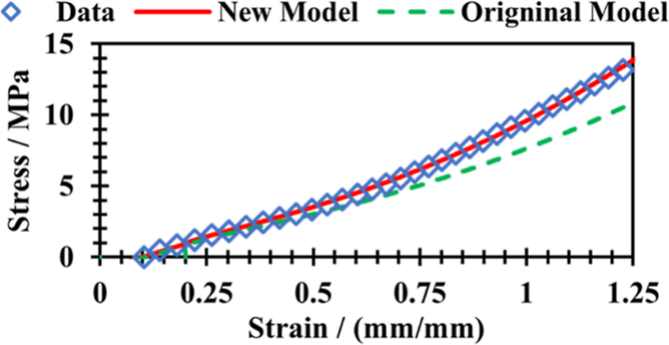

A new model is presented to predict rubber behavior during chemical aging at fixed strains. The model is validated using a carbon black-filled nitrile butadiene rubber aged in air at 125 °C. The model improves upon Tobolsky’s dual network theory, designed for unfilled elastomers undergoing conventional aging but which has also often been used in rubber composites undergoing more complex aging scenarios. This work explores the shortcomings of the original model and demonstrates how the new model overcomes them. The model was validated using uniaxial tensile samples aged at 125 °C for 24–72 h at strains from 0–30%. The permanent set was measured, and the samples were tested on an Instron uniaxial test machine after aging. The cross-link density was estimated by equilibrium swelling. Results show that the new model more accurately models the stress–strain behavior to higher strains and provides more reliable estimates of chain scission and cross-linking after aging.

## Introduction

### Chemical Stress Relaxation

**Figure 1:**
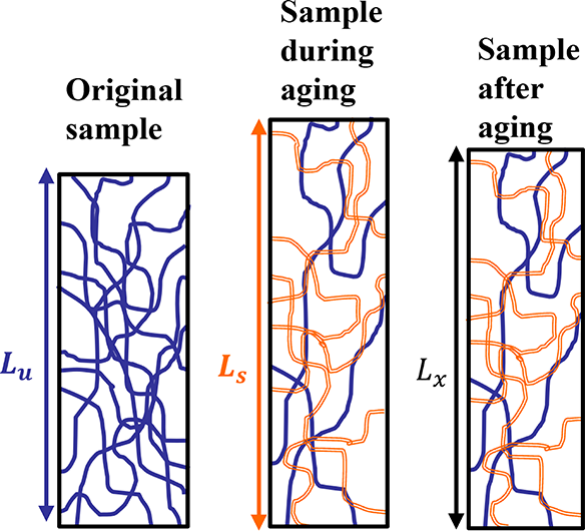
Diagram illustrating the breakdown of the original network and the formation of a new network (hollow orange).

When rubber samples are held at a set strain at an elevated temperature, their stress in the direction of the strain begins to relax. Upon release, the rubber does not return to its original length and instead exhibits a permanent set.^1,2^ Permanent set and chemical stress relaxation are huge concerns in a variety of rubber applications. In soft rubber and polyurethane tires, a large permanent set can lead to flat spots that increase noise and reduce fuel efficiency.^3^ Permanent sets can negatively impact rubber seals.^4,5^ Chemical stress relaxation in rubber vibration dampers can alter their ability to work effectively.^6,7^ The process of chemical stress relaxation, studied by Tobolsky and co-workers,^2,8–10^ is distinct from viscoelastic stress relaxation and is called chemical stress relaxation. Tobolsky put forward a model that is still widely used and tested.^11–14^ Tobolsky proposed that chemical stress relaxation was the consequence of chemical reactions taking place in the rubber molecules that break and reform cross-links in the polymer network. The rate of these reactions (chain scissioning and cross-linking) has been shown to increase with oxygen and heat,^15^ and this is reflected in an increased chemical stress relaxation rate.^2^ Both chain scission and cross-linking happen simultaneously and at different rates. The dual-network theory suggests that the results of both chemical reactions can be treated as a breakdown of the original network and the simultaneous creation of a new network in the deformed configuration. These networks are treated as separate, acting in parallel with no interactions, which is an oversimplification.^16–19^ The original network is in equilibrium with the original unstrained stretch, while the new network is in equilibrium with the stretch at which it was formed. Figure 1 shows a schematic of this process.

In Figure 1, the sample that has length *L*_u_ is initially at rest. It is composed entirely of the original, as molded network, represented by the solid blue lines. The sample is then stretched to length *L*_s_ and held at an elevated temperature. By the end of the experiment, the original network had diminished, while a new network represented with hollow orange lines had been formed. Following the test, the sample retains the two networks, which, at any length *L*_x_ will have different extension ratios. The stress at length *L*_x_ can be calculated by using the dual network theory. Permanent set can be found by calculating the length *L*_x_ that results in zero stress. This value of *L*_x_, which results in zero stress, will be between the lengths *L*_u_ and *L*_s_, meaning that the original network will be in tension and the new network will be in compression with these two forces being balanced.

### Dual Network Theory

A simple model based on Tobolsky’s version of the dual-network theory for elastomers with no filler reinforcement is presented. The model assumes that after aging, there are two rubber networks acting in parallel, which are independent of each other. Each network is in equilibrium with the length at which it was formed, and its contribution to stress is proportional to its network density. The total stress at length *L*_x_ is represented by eq 1, where the first term represents the contribution of the original network, and the second term represents the contribution of the new network.
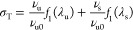
1where
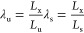
σ_T_ is the total stress at length *L*_x_*,* and ν_u0_, ν_u_, and ν_s_ are the network density of the original network before aging, the original network after aging, and the new network after aging, respectively. λ_u_ and λ_s_ are the extension ratios of the original and new networks, respectively. *L*_u_, *L*_s_, and *L*_x_ are the lengths of the sample before aging, during aging, and at the stress σ_T_, respectively.

In eq 1, the first term represents the contribution of the original network, and the second term represents the contribution of the new network. In both terms, the network density of each network is multiplied by a function of the extension ratio, but crucially, the extension ratio is defined differently for each term. Since the original network is in equilibrium with the sample before aging, its extension ratio is the ratio of the sample length during testing and the original length of the sample before aging. The second term defines the extension ratio as the ratio of the test length divided by the length the sample was aged at. *f*_1_(λ) can take many forms; one commonly used form is that shown in eq 2
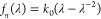
2where *k*_0_ is a constant. Equation 2 is developed by assuming the simplest Neo-Hookean strain energy function. Considering the stress in the direction of strain for a sample in simple tension, the Neo-Hookean strain energy function returns eq 3
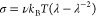
3where ν is the network density of the rubber sample, *k*_B_ is the Boltzmann’s constant, and *T* is the absolute temperature.^20,21^

Equation 4 is derived by substituting eq 2 into eq 1 and has been used in the past to describe the behavior of unfilled elastomers.^2,8–10^
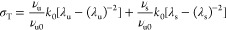
4However, for carbon black-filled nitrile butadiene rubber (NBR), the strain amplification effect caused by the presence of carbon black must be considered. Mullins & Tobin (1965)^22^ first introduced the concept of strain amplification and proposed eq 5 to describe the local amplified strain in a filled rubber sample.
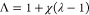
5where Λ is the amplified stretch ratio, λ is the applied stretch ratio, and χ is a constant called the strain amplification factor. Dinari et al. (2021)^23^ and Duncan et al. (2022)^24^ showed that for carbon black-filled synthetic rubber samples, eq 4 can be modified by substituting Λ (the amplified stretch ratio) for λ to give good agreement with experimental results. The assumptions made by the dual network theory allow the use of any function that adequately describes the stress–strain behavior of the rubber sample to be used for *f*_1_(λ). This work adopts the generalized Yeoh strain energy function (SEF) developed by Hohenberger et al. (2019),^25^ to derive *f*_1_(λ). Equation 6 shows the stress strain relationship derived using the generalized Yeoh SEF for the case of simple tension.^25^
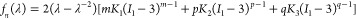
6where *I*_1_ = λ_1_^2^ + λ_2_^2^ + λ_3_^2^ is the first invariant of the stretch tensor and *m*, *K*_1_, *p*, *K*_2_, *q*, and *K*_3_ are user-defined parameters to fit the SEF to the stress–strain data.

## Test Method

### Materials

All tests were carried out on the same batch of carbon black-filled NBR compound. The most significant details of the formulation are listed in Table 1. The compound was mixed in an internal mixer and briefly masticated using a two-roll mill prior to being cured into 2 mm thick sheets using a hot press at 170 °C for 30 min.

**Table 1:** Formulation of the NBR Compound Used

component	details	loading pphr
NBR	(33% acrylonitrile content)	100
carbon black	N550	60
cross-link	sulfur	efficient vulcanization system

The NBR compound also contained the typical small amounts of plasticizer, activators, accelerators, and antioxidants that would be widely adopted in commercial practice.

### Sample Preparation

**Figure 2:**
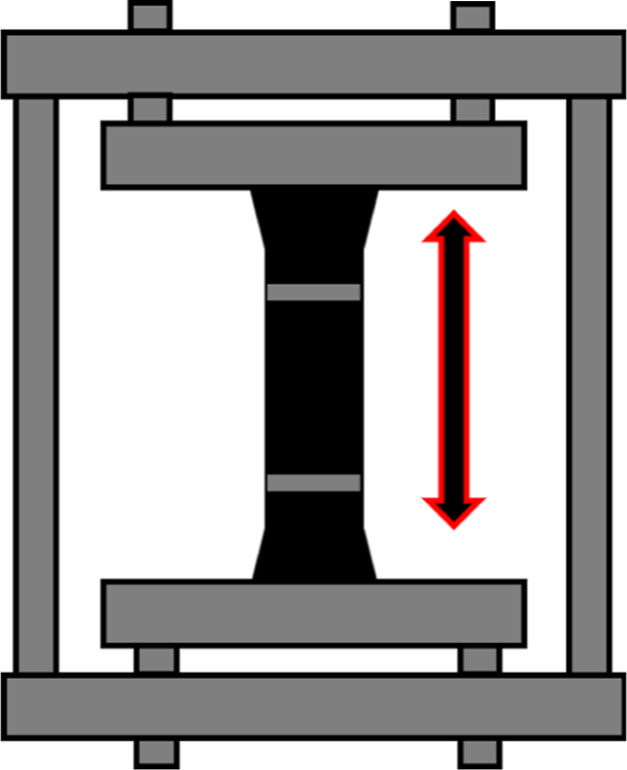
Simplified diagram of the stretched sample.

Dog-bone samples were cut out of the cured sheets using a cutter with dimensions specified in ASTM no. D412–16 (2021), die C, with a central cross-sectional area of approximately 2 mm × 6 mm. All samples were cut in the milling direction of the rubber sheets. Samples were gripped outside the gauge length in the stretching frame shown in Figure 2. Marks were placed on the rubber samples in the gauge length before the samples were placed in the stretching frame. These marks were used to ensure the samples were stretched to the correct strain before being placed in an oven at 125 °C. After aging, samples were removed from the oven and the stretching frame and left to cool overnight. The permanent set was measured and recorded using the marks introduced prior to aging, using a digital caliper.

### Tensile Testing

Samples were tested on a screw-driven Instron 5967 instrument using a video extensometer to track strain. All tests were done with a crosshead displacement of 1 mm/s, resulting in a strain rate of 1.5%/s. Samples were tested in tension from 0 to 100% strain.

### Cross-Link Density Measurements Using Equilibrium Swelling

Off-cuts were taken from aged tensile samples prior to testing. These samples were weighed and then left for 48 h in toluene in the dark. The toluene was then replaced with fresh toluene, and samples were left for a further 48 h, until they had reached equilibrium. The weight was then recorded again before the samples were left to dry out for 72 h again in the dark. They were weighed, left for a further 24 h, and weighed again to ensure that they had completely dried out. Finally, the density after swelling and drying of each sample was measured by using a density balance.

The Flory–Rehner theory^26^ was used to calculate cross-link density using the collected data and information from the recipe. This is done by applying eq 7
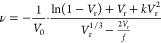
7where ν is the cross-link density, *f* is a factor depending on the functionality of cross-link (in this experiment, *f* = 4), *k* is the polymer–solvent interaction parameter (in this experiment, *k* = 0.505),^27^*V*_0_ is the solvent molar mass volume. Finally, *V*_r_ is the rubber fraction calculated using eq 8
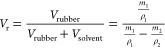
8where *m*_1_ is the mass of rubber,ρ_1_ is the density of rubber, *m*_2_ is the mass of absorbed solvent, and ρ_2_ is the density of absorbed solvent.

For filled materials such as those used in this work, the Flory–Rehner theory gives only an approximate value for cross-link density.^28–30^ However, as only one material is used, and only relative changes are needed to assess the impact of aging, it is appropriate.

## Aging Experiments

### Aging under No Strain

When aged under no strain, the original sample length and the length during aging are the same; therefore, λ_u_ = λ_s_. Therefore, eq 1 can be simplified to eq 9
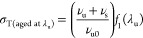
9

This implies that the change in sample stiffness, in the case of a sample aged under no strain, is proportional to the total change in cross-link density, with no other factors affecting the stiffness change during aging. To verify the validity of eq 9, some of the tensile samples were aged in air at 125 °C under no strain, and their change in cross-link density was measured using equilibrium swelling and then by applying the Flory–Rehner method using eq 7.

**Figure 3:**
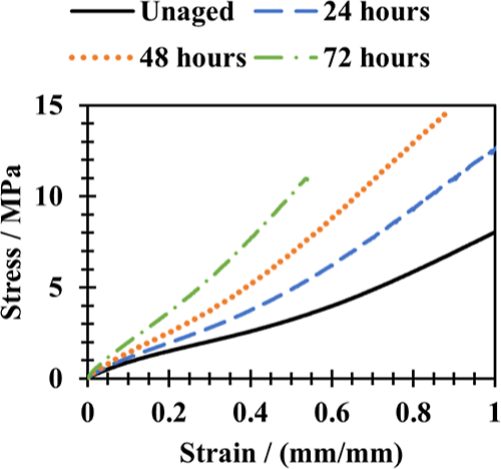
Stress–strain curve of the NBR sample before and after aging in air at 125 °C.

Figure 3 shows the stress–strain data from NBR samples aged under no strain for different durations (24, 48, and 72 h) in the air oven at 125 °C. This temperature was chosen as it is the upper end of the temperature range encountered in geothermal drilling applications. Table 2 shows the stress recorded at set strains for each sample. Table 3 displays changes to the cross-link density as measured using the Flory–Rehner method.

**Table 2:** Stress at 10, 30, and 50% Strain of NBR Samples Aged in Air at 125 °C

time aged	stress/MPa			stress/stress at same strain unaged		
/hours	at 10% strain	at 30% strain	at 50% strain	at 10% strain	at 30% strain	at 50% strain
0	0.90	2.04	3.23	1	1	1
24	1.14	2.79	4.89	1.27	1.37	1.51
48	1.40	3.74	6.88	1.58	1.83	2.13
72	2.00	5.50	10.10	2.22	2.69	3.12

**Table 3:** Change in Cross-Link Density of NBR Measured by Equilibrium Swelling and the Flory–Rehner Method

time aged (/hours)	cross-link density (/mol dm^–3^)	(ν_u_ + ν_s_)ν_u0_
0	0.000599	1.00
24	0.000749	1.25
48	0.000934	1.56
72	0.001252	2.09

According to eq 9, the change in stress at all strains should be proportional to the change in cross-link density. Tables 2 and 3 show that there is good agreement at low strains, for example, at 10% strain. However, this is not the case at higher strains. Samples aged for longer durations show the largest deviation. This result suggests that eq 9, which assumes that all the changes in the stiffness of the material are related to changes to the cross-link density, does not fully reflect the changes to the network. In other words, other factors have affected the stiffness of the material. Possible additional causes could include the loss of plasticizer. By comparing the sample weight before and after equilibrium swelling, it was revealed that all the aged samples leached out proportionally less plasticizer. This indicates that the aging process has resulted in some loss of plasticizer. It is also known that a reduced concentration of plasticizer will result in an increase in rubber stiffness at higher strains.^31–33^ Alternatively, a sooner onset of finite chain extensibility could also have been caused by the increased cross-link density or the fact that the network formed by oxidation may differ from that formed by sulfur vulcanization.^21^

### Change in Network Density

The dual network theory has been used in literature to calculate the rate of cross-linking and chain scission.^34,35^ This is achieved by comparing the cross-link density of the original network ν_u0_ to the surviving original network ν_u_ and the new network ν_s_. The network densities ν_u0_, ν_u_, and ν_s_ are found by using eq 1, and comparing data from unaged samples and samples aged under no strain and at a set strain.

**Figure 4:**
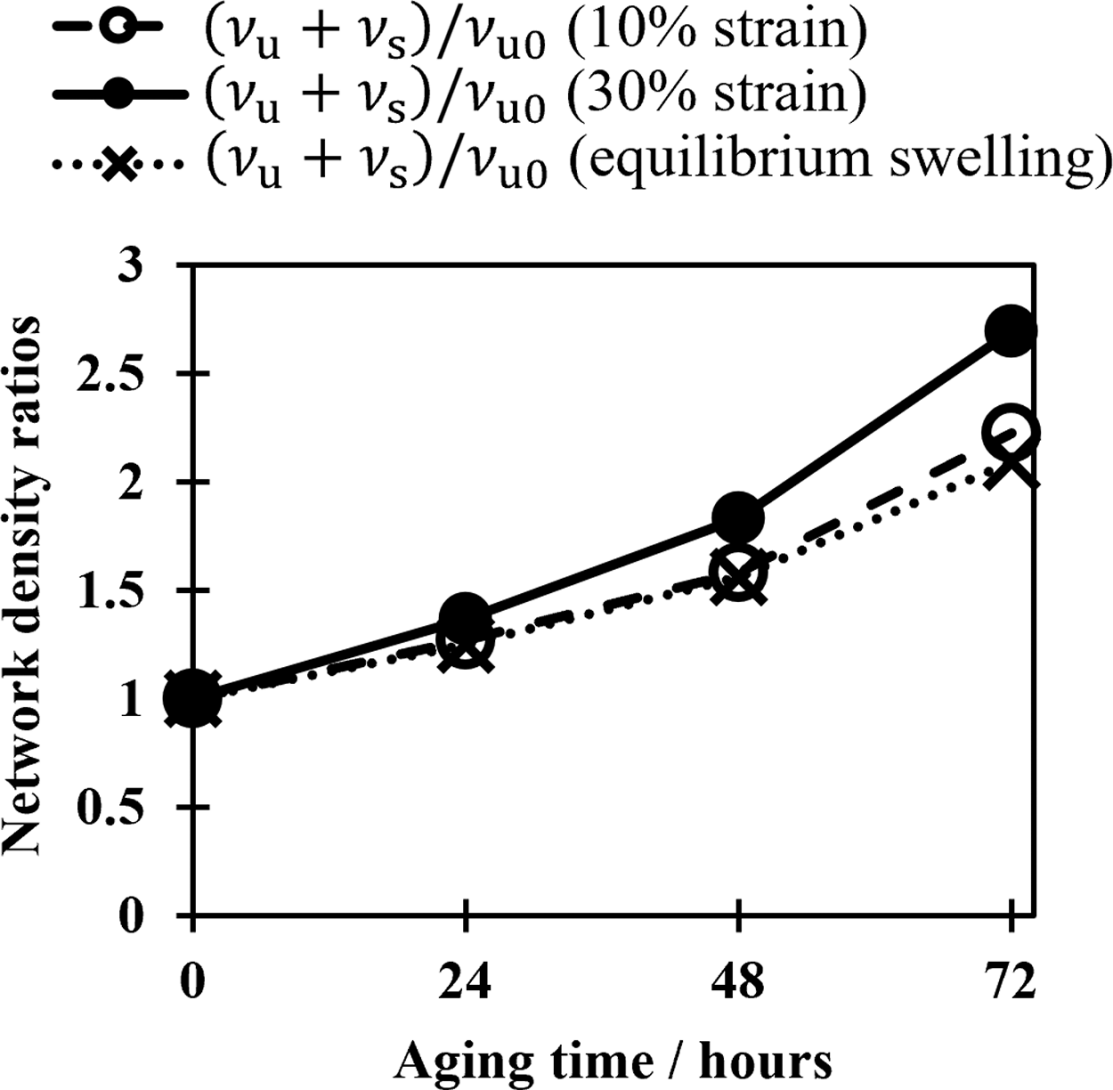
Network density ratios of the NBR sample as a function of time. Samples aged in air at 125 °C. Results calculated from samples aged under no strain, at 10% strain, and 30% strain using equilibrium swelling.

The total change in network density (ν_u_ + ν_s_)/ν_u0_ can be found by dividing eq 9 by *f*_1_(ν_u_). This is equivalent to a ratio of the stress of an aged sample with that of an unaged sample. Figure 4 shows how results found using this calculation considering stress at 10 and 30% strain are compared to results found using equilibrium swelling.

Figure 4 demonstrates that the ratio between the cross-link density before and after aging, as calculated using the dual network theory, depends heavily on the strain used to determine the ratio. When a 10% strain is used, the results are in good agreement with those obtained from equilibrium swelling, whereas when a strain of 30% is used, the dual network theory overpredicts the change in cross-link density. Figure 4 shows that at a 10% strain, the assumption that changes in cross-link density are proportional to changes in stiffness appears to be valid. However, this assumption is not true at larger strains, for example, at a 30% strain, leading to a calculated network density before and after aging that is higher than that obtained from the equilibrium swelling measurement. Since the dual network model assumes that all changes in stiffness are due to increased network density, any other factors that increase stiffness result in a higher calculated cross-link density than that observed.

Another ratio to consider is ν_s_/ν_u_. Calculating ν_s_/ν_u_ does not require any data from an unaged sample. We can determine ν_s_/ν_u_ by comparing the stress of a sample aged at no strain to that of a sample aged under constant strain at the strain at which it is aged. Since the new network is in equilibrium with the strain at which it is formed, it will have no contribution to the stress of the sample aged under strain when held at the strain it is aged at. Therefore, the value of *f*_1_(λ_s_) from eq 1 becomes zero when the sample is at a stretch of λ_s_. As a result, eq 1 simplifies to eq 10. Dividing eq 9 by eq 10 gives eq 11
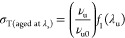
10
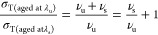
11therefore
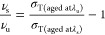
12

**Figure 5:**
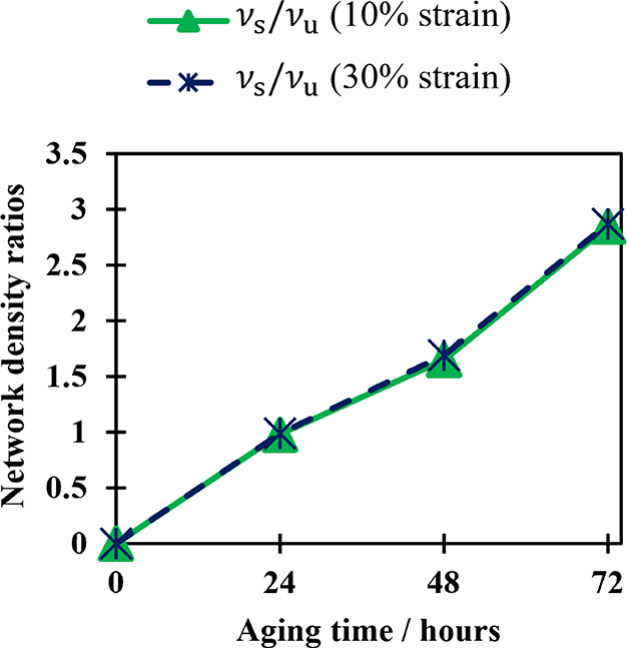
Network density ratios of the new network to the surviving original network ν_s_/ν_u_ for an NBR sample as a function of time. Samples aged in air at 125 °C. Results calculated from samples aged under no strain and at 10% strain and 30% strain.

The ratio of new network density to original network density (ν_s_/ν_u_) at 10% strain and 30% strain was found using data from samples aged under no strain and samples aged at 10 and 30% strain, respectively. As Figure 5 shows, using either 10 or 30% strain gives very similar results.

The ratio of the new network to the surviving original network (ν_s_/ν_u_) is in close agreement, whether it is found using a sample aged at 10% strain or 30% strain. This is because it does not rely on data from unaged samples, meaning that all samples have undergone the same aging processes. Therefore, additional factors beyond chemical aging do not affect the result.

Both ratios calculated in this section, ν_s_/ν_u_ and (ν_u_ + ν_s_)/ν_u0,_ are used to determine the variables used in dual network theory, as shown in eq 1, ν_u_/ν_u0_ and ν_s_/ν_u0_. It is therefore a large concern that the value calculated for (ν_u_ + ν_s_)/ν_u0_ is, in our case, very dependent on the strain considered for that calculation, and that for strains larger than 10%, this value disagrees with experimental values calculated from the equilibrium swelling measurements of cross-link density. This obviously means that predictions of stress–strain values made using the model cannot be accurate all the time, as they will differ depending on which strain is chosen to calculate the variables. Many studies using the dual network theory only consider, or test, samples aged at one strain and do not confirm results using any other method.^34,35^ Those that have^19^ do find differences between the cross-link density found using Tobolsky’s dual network theory and that found using equilibrium swelling. We do see, however, that the value of (ν_s_/ν_u_)calculated does not depend on the strain used. Although no alternative method is used to verify the values calculated, this suggests that this value is more trustworthy than the values calculated for ((ν_u_ + ν_s_)/ν_u0_). A model built only using this variable would, in this case, not be dependent on the strain used to calculate its variables. This new model would also have the added benefit of not requiring any data from unaged samples.

## New Dual Network Model

The original dual network theory relies on data from samples before and after aging. As we have shown, the model can only account for chemical aging and is unable to account for other factors that cause differences in stiffness between unaged and aged samples. In this work, a new model that does not rely on data from an unaged sample is presented. The model is described using eq 13
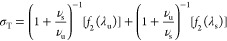
13where *f*_2_(λ_u_) is the stress–strain function of a sample aged under no strain.

Equation 13 is derived from eq 1 and eq 9. Beginning with eq 9, making ν_u0_ the subject, see eq 14. Equation 14 is then substituted into eq 1, see eq 15. Finally, σ_T(aged at λu)_ is replaced with *f*_2_(λ_u_) to give eq 13. Equation 13 draws many similarities to eq 1. In both cases, the first and second terms represent the contribution of the original and new networks after aging. And in both cases, a stress function is multiplied by an expression representing the amount of the original or new network after aging. The key difference is that the new model requires no input from the unaged sample. The stress function adopted in this case is taken from a sample aged under no strain. This means that factors other than chemical aging that cause differences between unaged and aged samples have no impact on the results generated by the model. The model relies on the assumption that the other factors affecting aging are unaffected by the strain of the sample during aging. Figure 5 shows that this assumption is true for the NBR samples used in this study. The figure shows that whether aging at 10% or 30% strain, there is a near-identical change in the cross-link density.
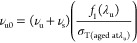
14
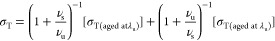
15

The network density ratio, ν_s_/ν_u_, can be determined in much the same way as in the original model by comparing the stress of a sample aged at no strain to a sample aged under constant strain at the strain at which it is aged. Since the new network is in equilibrium with the strain at which it is formed, it will have no contribution to the stress of the sample aged under strain when it is held at the strain it is aged at. As a result, eq 13 simplifies to eq 16.
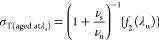
16Equation 16 can be rearranged to find ν_s_/ν_u_
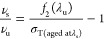
17

Since *f*_2_(λ_u_) is defined as the stress–strain function of a sample aged under no strain, this is the exact same method used for calculating ν_s_/ν_u_ that is used in the original model. Therefore, both models return the same value for ν_s_/ν_u_.

## Comparison of the Results

The following comparisons are made using the original dual network model using eq 1, where *f*_2_(λ) is a generalized Yeoh model fit using eq 6, to stress–strain data from an unaged sample. The parameter values and fit for the generalized Yeoh model are shown in Table S1 and Figure S1. This model is compared to the new multifactor dual network model using eq 13 where *f*_2_(λ) is a generalized Yeoh model using eq 6 that is fit this time to stress–strain data of a sample aged under no strain for 24 h at 125 °C. The parameter values and fit for this model are also shown in Table S1 and Figure S1.

### Modeling Stress–Strain

**Figure 6:**
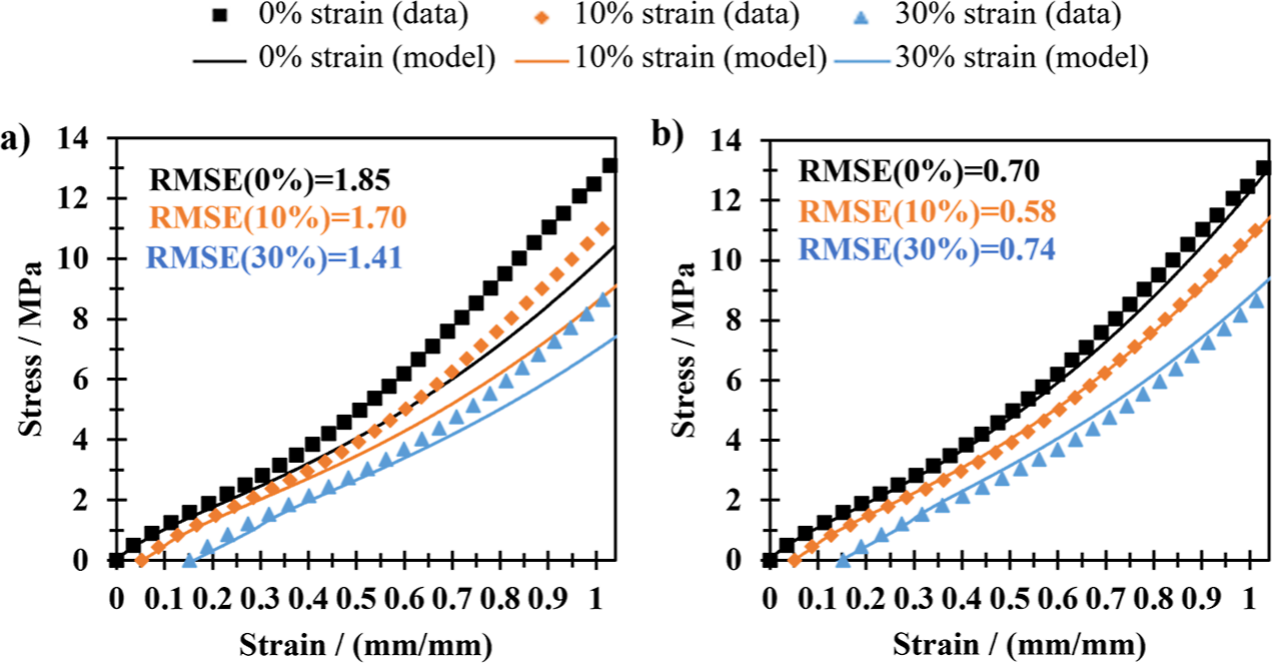
Stress–strain plot of NBR samples aged at fixed strains for 24 h at 125 °C. Left (a) shows the model based on the original dual network model; right (b) shows the model based on the new dual network model. The RMSE for each fit is shown.

Results from samples aged at 0, 10, 15, 20, 25, and 30% strains for 24 h at 125 °C are shown in Figure S2. To simplify the figure, Figure 6 shows only the stress–strain data from samples aged for 24 h at 125 °C at rest and at 10% and 30% tensile strain. Unsurprisingly, the new model, which uses data from a sample aged under no strain, has a good fit to this sample. Both models make good predictions for permanent sets. The original model does a poor job of predicting stress–strain behavior at higher strains. Figure 6 shows the root-mean-square error (RMSE) for all fits.

### Permanent Set

Permanent set is found using both models by setting σ_T_ = 0 and solving for λ_u_.

**Figure 7:**
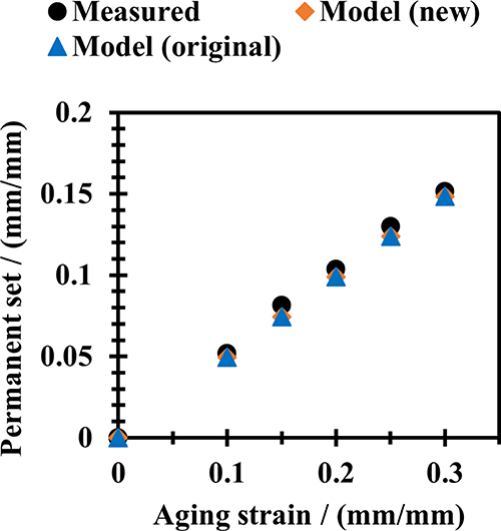
Permanent set after aging NBR samples at a fixed strain for 24 h at 125 °C.

Figure 7 shows the permanent set measured after aging samples at 10, 15, 20, 25, and 30% tensile strain for 24 h at 125 °C. The original dual network model gives an accurate prediction for permanent set values. This was expected, as the original dual network model is commonly used for these types of prediction.^10,36^ The original dual network model’s ability to make good predictions on a permanent set despite its poor prediction on stress–strain data has rarely been commented on. In this work, it is believed that factors that cause a changing stiffness in tension can have the same effect in compression, meaning that the balance between the original network (which is in tension at rest) and the new network (which is in compression) is not strongly affected by these factors. We see from Figure 7 that the new model, like the original model, makes a good prediction of the permanent set.

## Discussion

### Evaluating the Independent Network Assumption

The dual network theory, as put forward by Tobolsky, often struggles to predict the stress–strain behavior of samples where factors other than changes in cross-link density affect stiffness. In particular, the theory encounters difficulties in predicting higher strain values. A commonly targeted flaw in dual network theory is the assumption that the two networks act independently of each other. Models have been developed to compensate for this oversimplification.^16–19^ However, this work demonstrates that the additional complexity introduced by these models may be unnecessary. By considering the other aging factors and utilizing the new model proposed in this study, stress–strain values, including those at higher strains, can be significantly improved. Although the assumption of independence between the two networks is an oversimplification, the findings presented here indicate that it is nevertheless an appropriate simplification.

### Estimating the Cross-Linking and Chain Scission Rate

Researchers have utilized the dual network theory to estimate the change in cross-link density, represented by ν_u0_/(ν_u_ + ν_s_), as well as the rate of cross-linking and chain scission.^34,37,38^ This estimation can be accomplished by subjecting a sample to aging at a fixed strain and monitoring its stress-relaxation. These results are then compared to those of a sample aged without strain.^34^ However, the findings illustrated in Figure 4 highlight the problematic nature of this approach. When the experiment is repeated at different fixed aging strains, it may yield varying outcomes.

For more reliable results, this experiment should be repeated at multiple aging strains; the change in cross-link density can then be extrapolated to zero strain. If possible, the experiment should be repeated in both tension and compression, and the change in cross-link density can then be interpolated to zero strain.

Alternatively, Figure 5 demonstrates that the ratio of the new to original network, ν_s_/ν_u_, remains consistent regardless of the strain applied. This ratio, in conjunction with other techniques for calculating changes in cross-link density, such as equilibrium swelling, should be employed to yield more reliable estimations of the rate of cross-linking and chain scission.

### Factoring out Cross-Link Density Changes

**Figure 8:**
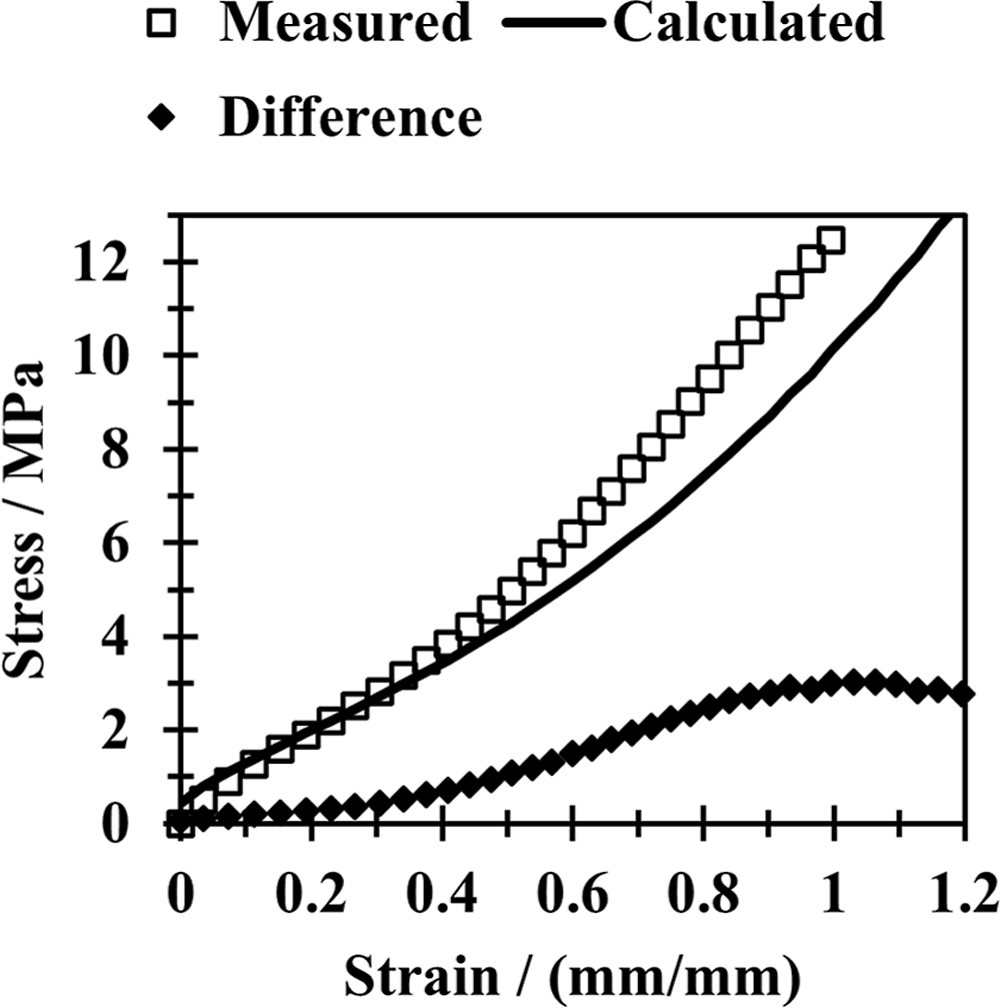
Stress–strain plot of an NBR sample aged at no strain for 24 h at 125 °C. Calculated values found by multiplying the stress values of an unaged sample by the increase in cross-link density caused by 24 h of aging at 125 °C.

Aging of rubber nearly always results in a change in the cross-link density. This makes it very difficult to investigate and isolate additional mechanisms that also take place during aging. This work demonstrates that, for the material used, changes in modulus are not only the consequence of changes in cross-link density. Figure 8 highlights this point by showing the stress–strain data found for an NBR sample aged for 24 h at 125 °C under no strain. Also shown are the stress–strain values that would result from multiplying the stress values of an unaged sample by the increase in cross-link density caused by 24 h of aging at 125 °C. To make this calculation, the change is related to that found from the equilibrium swelling data in Table 3. The difference between the two, also plotted in Figure 8, is proposed to result from additional aging factors, such as a loss of plasticizer.

**Figure 9:**
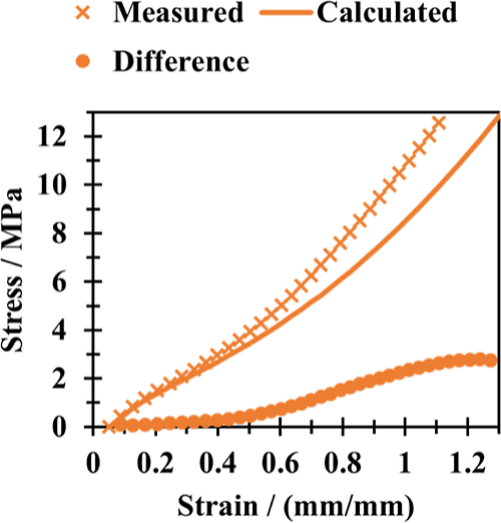
Stress–strain plot of NBR samples aged at 10% strain for 24 h at 125 °C. Calculated values calculated using the original dual network model.

Applying the same method to a sample aged under strain becomes even more complex. The original dual network model assumes that all changes in stiffness stem from variations in cross-link density. Consequently, data from an aged sample can be compared to the stress–strain values predicted by the original dual network model, similar to the method used for the sample aged under no strain. However, as demonstrated in this work, the original dual network model fails to produce reliable estimates of the change in cross-link density, ν_u0_/(ν_u_ + ν_s_). Nevertheless, as mentioned previously, the ratio ν_s_/ν_u_ can be combined with other techniques to calculate changes in cross-link density, which can yield a more reliable outcome. By utilizing these values to compute the variables in the original dual network model, comparisons between those results and the experimental data of a real sample can be utilized to assess the impact of other aging factors. Figure 9 shows the stress–strain data from a sample aged at 10% strain for 24 h at 125 °C, along with the stress–strain values generated by the original dual network model.

Both Figures 8 and 9 demonstrate that additional aging factors such as the loss of plasticizers have a significant impact on samples aged under strained and unstrained conditions. A slight increase in stiffness is observed at lower strains. Then, at the onset of finite chain extensibility, as indicated by an increase in modulus at higher strains,^21^ the contribution of plasticizer loss becomes more substantial before reaching a plateau. These findings highlight that even when utilizing data solely from a sample aged at a fixed strain, it remains feasible to factor out the influence of changes in cross-link density and draw similar conclusions to those obtained from data collected for a sample aged without strain.

## Conclusions

As the dual network model was originally developed for simple aging scenarios on unfilled elastomers, it is only capable of accounting for chemical aging and is unable to account for other factors that cause differences in stiffness between unaged and aged samples. In this work, a new model was presented based on the same principles as Tobolsky’s original model, but that does not rely on data from an unaged sample. This means that factors other than chemical aging that cause differences between unaged and aged samples will not have an impact on the results generated by the model. Both models were tested using tensile samples of carbon black-filled NBR aged at 125 °C for 24–72 h at strains from 0–30%. The new model: requires less data and relies on fewer variables; is better at modeling the stress–strain of aged samples; does not lead to false conclusions on the rate of cross-linking and chain scission, unlike the original model; and, like the original model, provides accurate predictions of the permanent set.

## Supporting Information Available

Parameters and figures showing the generalized Yeoh fit of NBR material unaged and after aging at 0% strain, and comparison of the original dual network model and new model fits for all aging strains (PDF)
