# Supplementary material for: Improved Dual Network Model for Aging of Rubber Composites under Set Strains
Source: Macromolecules. 2023 Aug 22;56(17):6704–11. doi: 10.1021/acs.macromol.3c01131 (PMC10501203; doi:10.1021/acs.macromol.3c01131)
Supplement: Supplementary file 1 — ma3c01131_si_001.pdf [file ma3c01131_si_001.pdf]

Supporting Information for "Improved Dual Network Model for Ageing of Rubber Composites Under Set Strains"

Aaron M. Duncan[1]‡, Keizo Akutagawa[1], Julien L. Ramier[2], James J.C. Busfield[1]

[1]School of Engineering and Material Science, Queen Mary University of London, Mile End Road, London, United Kingdom, E1 4NS

[2]SLB Cambridge Research, Cambridge, United Kingdom, CB3 0EL

I. Generalized Yeoh model fits

Table S1. Parameters used with eq 5 to model NBR material.

Parameter	Value for unaged material	Value for material aged under no strain of 24 hours
m	0.93	0.92
K_1	1.40	1.76
p	1.40	1.25
K_2	-0.50	-0.50
q	1.72	1.70
K_3	0.70	1.05

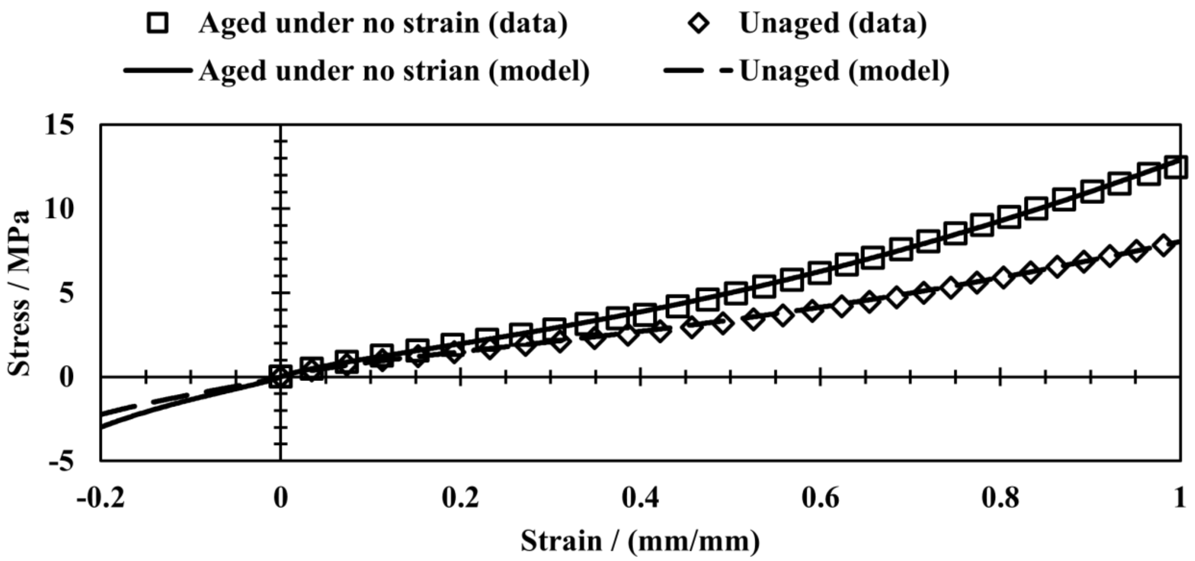

Figure S1. Stress-strain data from unaged samples and samples aged under no strain. Model based on generalized Yeoh strain energy function.

II. New and old model comparison

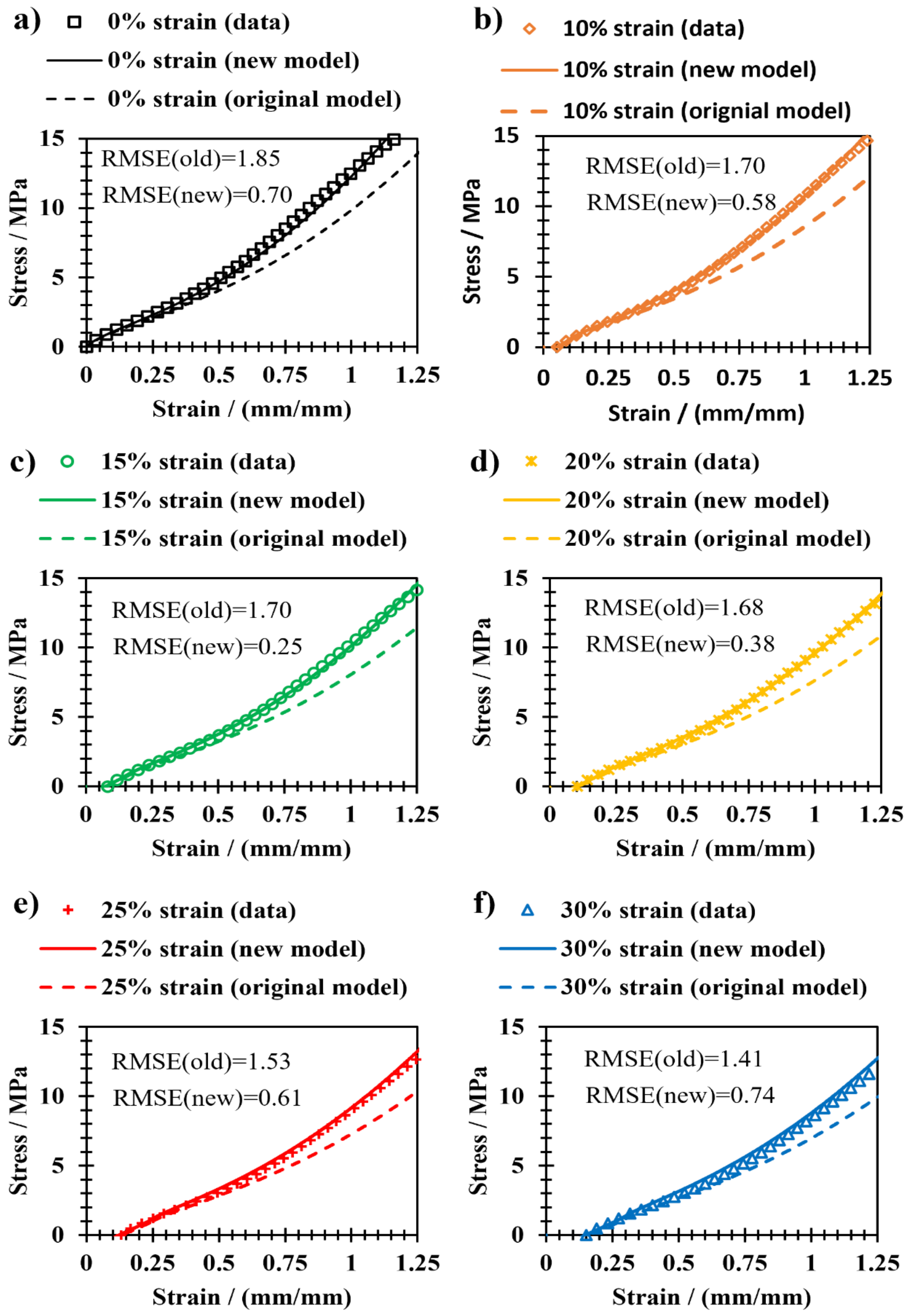

Figure S2. Stress-strain plot of NBR samples aged at fixed strains for 24 hours at 125℃. Samples aged at 0, 10, 15, 20, 25, and 30% strain. Each figure shows the original dual network model (dashed lines) and the new dual network model (solid lines).
